# A Fatal Cause of Rapidly Progressive Heart Failure in a Middle-Aged Woman

**DOI:** 10.7759/cureus.21401

**Published:** 2022-01-19

**Authors:** Tatiana Salazar, Isabel Freitas, Carla Melo, Anabela Ferreira, Luisa Guerreiro

**Affiliations:** 1 Internal Medicine, Centro Hospitalar Do Médio Ave, Vila Nova de Famalicão, PRT; 2 Internal Medicine, Centro Hospitalar do Médio Ave, Vila Nova de Famalicão, PRT; 3 Internal Medicine Service, Hospital Pedro Hispano - Matosinhos Local Health Unit, Matosinhos, PRT

**Keywords:** cardiothoracic surgery, heart failure, intimal sarcomas, sarcomas, primary malignant tumors

## Abstract

Primary cardiac neoplasms are rare, with 3/4 cases being benign. Most malignant neoplasms are sarcomas. Clinically, they present as pseudovalvular obstruction or remote embolism and rarely as a paraneoplastic syndrome. Median survival depends on complete resection rather than histologic type. We describe the case of a 65-year-old woman who presented to the hospital with a three-month history of asthenia, anorexia, weight loss, and progressive worsening of exertional dyspnea. Transthoracic echocardiogram showed a bulky mass in the auricles with significant transvalvular obstruction of the mitral valve. The CT scan showed a voluminous mass in the interauricular septum with the invasion of both atria and restriction of flow in both pulmonary veins. A transvenous biopsy was performed and histology revealed a primary intimal sarcoma. The patient was not eligible for surgery and was proposed for palliative chemotherapy, but she succumbed to her illness in less than two weeks. This report describes this rare and rapidly fatal disease and reviews the literature.

## Introduction

Cardiac tumors may be primary or secondary, the latter being 100 to 1000 times more common than the former [1]. The pulmonary source is the most common primary location leading to metastatic cardiac involvement [2]. Primary cardiac tumors with an incidence of 0.001 to 0.03% based on autopsy findings are uncommon [3]. Benign primary cardiac tumors account for nearly 75% and almost half are myxomas. 25% of primary cardiac tumors are malignant, and among these, sarcomas predominate, while lymphomas are second (about 5%) [4]. Intimal sarcomas are rare and occur more commonly in large arterial vessels, but are particularly unusual in the heart [5-7]. Here we describe a peculiar presentation of this rare entity with a rapidly fatal course.

## Case presentation

A 65-year-old woman with dyslipidemia and a history of obesity presented to the emergency department. For the past three months, she had complained of worsening asthenia, anorexia, weight loss, and dyspnea on exertion. She had been to the emergency department several times and was receiving beta-blockers and anticoagulants because she was suffering from new-onset atrial fibrillation (AF). On physical examination, she had a normal body temperature, a heartbeats in the normal range, and a normal blood pressure in the supine position. The other cardiovascular findings were unremarkable; she had crackles in the pulmonary base on auscultation, peripheral blood O_2_ saturation was normal on room air, and had pretibial edema of both limbs. Abdominal and neurologic examinations were normal. Laboratory examination showed anemia (hemoglobin of 9.9 g/dl; reference range: 12-16 g/dl), thrombocytopenia (125,000/µl platelets; reference range > 145.000/µl), elevated liver parameters (total bilirubin 1.8 mg/dl; reference range: < 1.0 mg/dl and DHL 780 UI/l; reference range: 100-350 UI/l) and elevation of N-terminal part of natriuretic peptide type B (1128.9 pg/ml; reference range < 100 pg/ml). The other tests were normal. An electrocardiogram showed sinus rhythm and a normal rate of 70 beats per minute. The patient was admitted with new-onset heart failure for evaluation and investigation.

**Figure 1:**
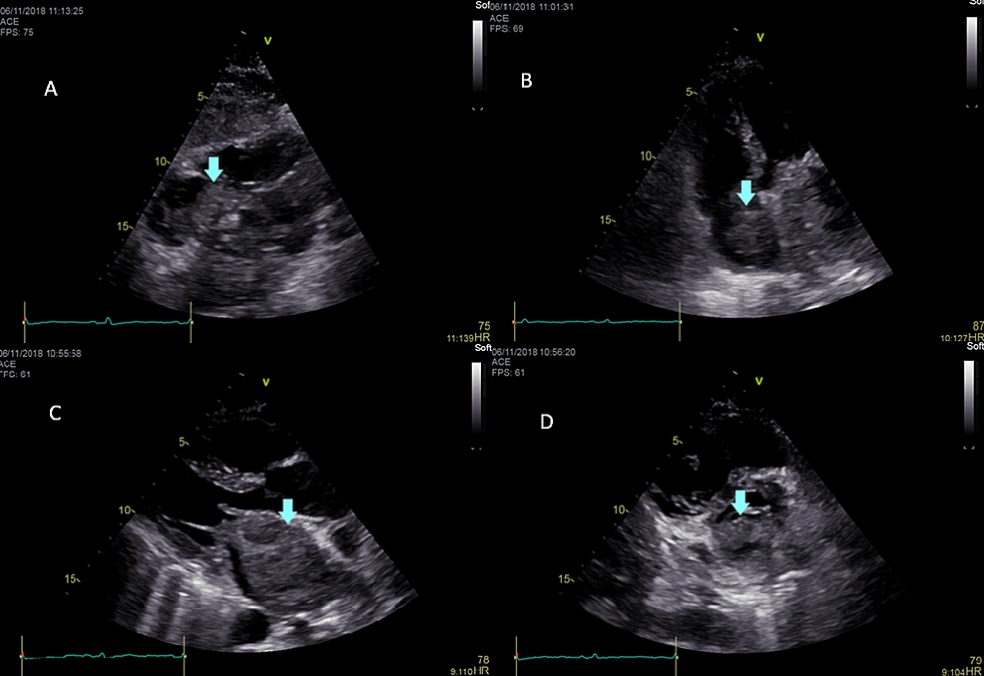
Transthoracic echocardiogram of the left atrium showing a voluminous mass infiltrating the atrial septum and extending into the right atrium (blue arrows). (A) Subcostal four-chamber view, (B) apical four-chamber view, (C) parasternal short-axis view, and (D) parasternal long-axis view.

During hospitalization, the patient re-entered AF and required rate control with beta-blockers. Despite adjustment of diuretics, acute hypoxemic respiratory failure was observed. To better characterize cardiac morphology and functionality, an echocardiogram was performed, which showed a mass with voluminous dimensions greater than 62 × 53 mm at the level of the left atrium, resulting in significant obstruction, with a mean gradient between the left atrium and left ventricle (LA/LV) of 18 mmHg, and which appeared to have infiltrated the atrial septum and extended to the right atrium without causing obstruction but with indirect evidence of right ventricular overload (Figure 1).

**Figure 2:**
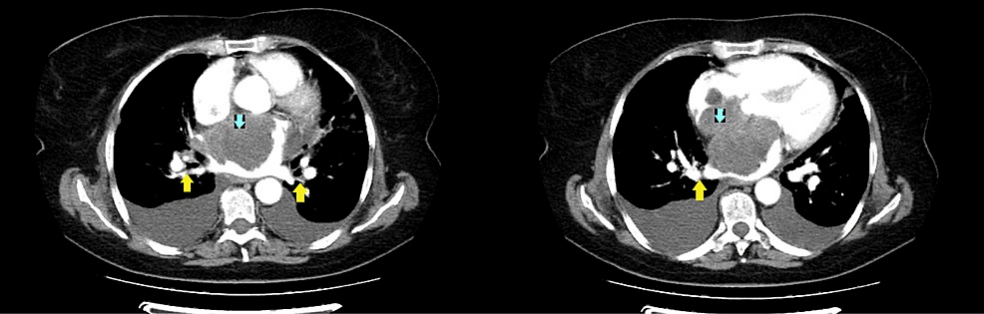
Thorax CT scan with contrast (transverse section) showing a tumor in the left atrium (blue arrows). The tumor is compressing the pulmonary veins (yellow arrows).

The CT contrast study showed a mass in the left atrium with lobulated contours and invasion of the intra-auricular septum and right atrium, restricting normal vascular flow between the lung and left atrium and partially and indirectly blocking pulmonary venous blood flow (Figure 2).

**Figure 3:**
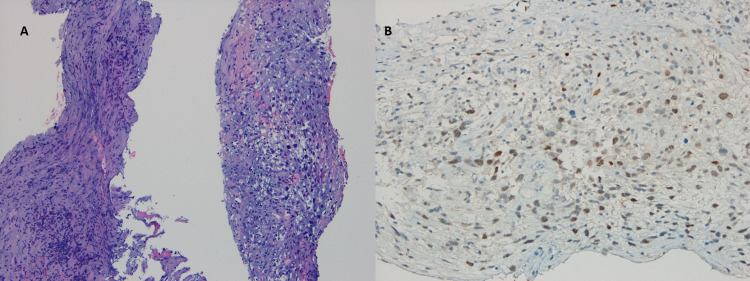
Malignant mesenchymal neoplasm composed of spindle-shaped and epithelioid cells (A) with multifocal positivity for MDM2 (B). MDM2: mouse double minute 2 homolog

Histology of the transvenous mass biopsy revealed a malignant mesenchymal neoplasm composed of spindle-shaped and epithelioid cells. Immunohistochemistry showed multifocal positivity for a murine double minute two (MDM2). Expression of CD34, cytokeratin (antigen E1/antigen E3), and desmin were negative, supporting a diagnosis of intimal sarcoma (Figure 3).

After evaluation by the cardiothoracic team, the patient was not eligible for surgical resection and palliative chemotherapy with doxorubicin, and ifosfamide was suggested to her. While she was being prepared for this, clinical deterioration occurred and within two weeks the patient died of cardiogenic shock due to severe left ventricular flow obstruction.

## Discussion

Primary cardiac sarcomas can occur at any age but are most common in middle age, between 30 and 50 years of age [7], with a male to female ratio of approximately one to one [1]. The most common cardiac sarcoma is angiosarcoma [1]. Spindle cell sarcomas are rare mesenchymal tumors that mostly occur in large arterial blood vessels, but are rare primarily in the heart [6-9]. They involve the pulmonary artery more than the aorta and most commonly the pulmonary trunk. Histologically, these tumors consist of atypical spindle cells with varying degrees of atypia and may have large myxoid areas. When located in the heart, the differential diagnosis includes angiosarcomas, the former usually showing positive immunoreactivity for MDM2, osteopontin, and vimentin and variable positivity for desmin [4].

Cardiac tumors may present with cardiac signs and symptoms due to intracardiac obstruction (in left-sided mass), systemic embolism (early metastasis in right-sided origin), or, less commonly, constitutional symptoms as a paraneoplastic syndrome. These tumors are diagnosed by transthoracic echocardiogram or CT examination [10].

Prognosis is as for other histologic types and depends mainly on complete surgical resection. These tumors are extremely destructive and the median survival time is little more than one year [6]. Definitive chemotherapy or radiotherapy alone has little prospect of rapidly relieving symptoms, so complete surgical resection is required. Incomplete resection usually leads to rapid local recurrence [11]. The most essential factor for prolonged survival is complete resection of the margins, although this is often not possible due to the highly destructive nature of the tumor, which often involves adjacent vital structures [12]. Although recurrences are very common, low-grade radiotherapy can be used as an adjuvant treatment option [10]. There are few cases of auto-transplantation or orthotropic heart transplantation worldwide. This option represents the solution with the greatest success in terms of complete resection, but further studies in this field are needed [11].

## Conclusions

In conclusion, early detection and treatment are of paramount importance because of their prognostic and beneficial therapeutic effects. Other, more common diseases present with these findings, so health care providers must be extremely suspicious, as symptoms may be nonspecific, and non-contrast CT scan may be non-diagnostic leading to delayed diagnosis. The final diagnosis was possible upon an abrupt clinical deterioration during hospitalization by executing a CT scan with contrast. Thus, reporting such cases is of utmost importance to raise clinical awareness of such aggressive, rapidly fatal tumors.
